# Mortality reduction and cumulative excess incidence (CEI) in the prostate-specific antigen (PSA) screening era

**DOI:** 10.1038/s41598-024-55859-z

**Published:** 2024-03-09

**Authors:** Patrick W. McLaughlin, Matthew M. Cousins, Alex Tsodikov, Payal D. Soni, Juanita M. Crook

**Affiliations:** 1 Department of Radiation Oncology, University of Michigan, Ann Arbor, MI USA; 2 Department of Radiation Oncology, Assarian Cancer Center, Ascension Providence Hospital, Novi, MI USA; 3 Department of Advanced Radiation Oncology, Self Regional Healthcare, Greenwood, SC USA; 4 Department of Biostatistics, University of Michigan School of Public Health, Ann Arbor, MI USA; 5 Department of Radiation Oncology, Dignity Health Cancer Institute, Phoenix, AZ USA; 6 British Columbia Cancer Agency and University of British Columbia, Kelowna, BC Canada

**Keywords:** Oncology, Cancer screening

## Abstract

The extent to which PSA screening is related to prostate cancer mortality reduction in the United States (US) is controversial. US Surveillance, Epidemiology, and End Results Program (SEER) data from 1980 to 2016 were examined to assess the relationship between prostate cancer mortality and cumulative excess incidence (CEI) in the PSA screening era and to clarify the impact of race on this relationship. CEI was considered as a surrogate for the intensity of prostate cancer screening with PSA testing and subsequent biopsy as appropriate. Data from 163,982,733 person-years diagnosed with 544,058 prostate cancers (9 registries, 9% of US population) were examined. Strong inverse linear relationships were noted between CEI and prostate cancer mortality, and 317,356 prostate cancer deaths were avoided. Eight regions of the US demonstrated prostate cancer mortality reduction of 46.0–63.7%. On a per population basis, the lives of more black men than white men were saved in three of four registries with sufficient black populations for comparison. Factor(s) independent of CEI (potential effects of treatment advances) explained 14.6% of the mortality benefit (p-value = 0.3357) while there was a significant main effect of CEI (effect = −0.0064; CI: [−0.0088, −0.0040]; p-value < 0.0001). Therefore, there is a strong relationship between CEI and prostate cancer mortality reduction that was not related to factors independent of screening utilization. Minority populations have experienced large mortality reductions in the context of PSA mass utilization.

## Introduction

With mass utilization of prostate-specific antigen (PSA) screening for prostate cancer beginning in the late 1980s, prostate cancer mortality and disease characteristics at presentation changed profoundly. Before the introduction of PSA testing, 20% of Caucasians and 40% of African Americans (AA) had metastatic disease at diagnosis^1,2^. After PSA screening was introduced, metastases at diagnosis declined, and within 10 years, annual mortality began to fall^2^. Randomized controlled trials sought to clarify the relationship between PSA screening and prostate cancer mortality in predominantly Caucasian populations^3^.

Two large randomized controlled trials had major impacts on policy and patient management^4,5^. The Prostate Lung Colorectal Ovarian (PLCO) study randomized 76,693 men to PSA screening or no screening and concluded that screening did not reduce mortality^6^. The European Randomized study of Screening for Prostate Cancer (ERSPC) randomized 162,387 men to PSA screening or no screening and found a 20% reduction in prostate cancer mortality in the screening arm^5^. Alternative explanations for mortality reduction seen with mass utilization of PSA screening, such as improved treatment, gained prominence^7–9^. Considering these trials, the U.S. Preventative Services Task Force (USPSTF) recommended against PSA screening in 2012.

The conclusion that PSA screening does not reduce prostate cancer mortality is under scrutiny. A follow-up analysis of the PLCO and ERSPC that addressed contamination and compliance suggested that mortality was indeed reduced by PSA screening^10,11^. USPSTF guidance was later modified in favor of selected screening in 2018^12^. Attention has been directed to other randomized trials, such as the ERSPC study components conducted in Goteborg and Rotterdam, where mortality reduction as high as 56% is reported^13,14^. Additionally, a lack of significant minority enrollment on major prostate cancer clinical trials (< 5%) in setting of lower quality prostate cancer outcomes has led to increasing interest in prostate cancer disparities^3^.

We tested the hypothesis that cumulative excess incidence (CEI), a measure of excess prostate cancer diagnoses (each diagnosis generally accomplished via PSA screening with associated biopsy if indicated), is related to reduced prostate cancer mortality through analysis of data from SEER in the US, using an approach distinct from that employed in the randomized controlled trials reviewed above. We also sought to determine through analysis whether there might be evidence of a potential effect of treatment advances on mortality reduction independent of CEI while also seeking to understand the impact of race.

## Methods

### Data sources

Data from the US on cancer incidence and mortality were sourced from the SEER Program (SEER; https://seer.cancer.gov/) for men aged 40–84 (smaller range for some regions), grouped in 5-year age cohorts from 1980 to 2016.

### Mortality, incidence, and screening utilization

**Figure 1:**
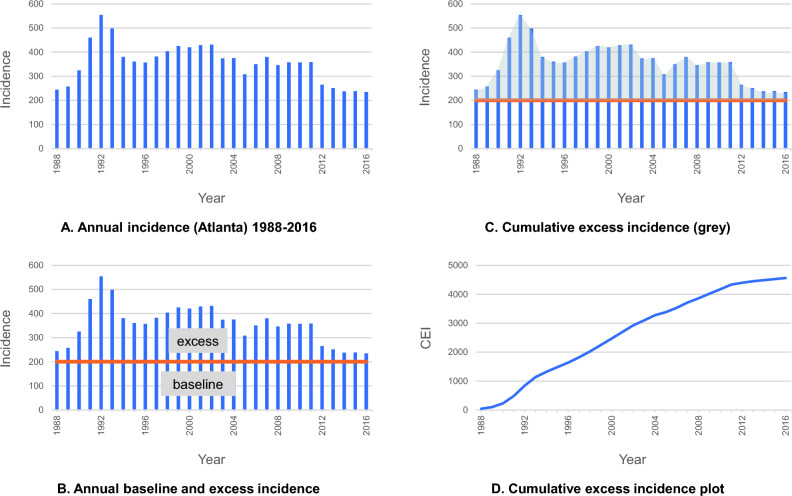
Derivation of cumulative excess incidence (CEI). The prostate cancer incidence for each year from 1988 to 2016 for a single SEER registry is depicted (**A**). The incidence for these years is divided into baseline incidence and excess incidence. Baseline incidence is the average of annual incidence values from 1980 to 1987 (orange horizontal line), and any incidence above this baseline is considered excess incidence for that year (**B**). The CEI for the year 2016 is depicted as the summation (shading) of excess incidence for 1988 to 2016 (**C**). The CEI is plotted over time from 1988 to 2016 and constitutes the summing of excess incidence from 1988 to each year from 1988 to 2016 (**D**).

Incidence and mortality were age-adjusted to the US male population (year 2000, ages 40–84) and standardized to rates per 100,000 person-years. All patients diagnosed with prostate cancer were included regardless of stage at diagnosis. Screening utilization was assessed using cumulative age-adjusted excess incidence (CEI) (Fig. 1; Supplementary Methods), a measure of the number of excess cases identified by PSA and biopsy above the established baseline incidence. Baseline incidence was defined as the average from pre-screening years (1980–1987). The entire United States was subject to PSA screening beginning in 1988. Excess incidence is the difference between the incidence in a given screening year (beginning in 1988) and the baseline incidence. CEI in a given year is the sum of annual excess incidence values during the period from the end of the pre-screening period to the year when CEI is calculated. Stated differently, the CEI can be determined by subtracting a curve for baseline incidence (average incidence 1980–1987) from a curve reflecting incidence over time since the PSA mass utilization. CEI is a surrogate for the cumulative screening utilization as of that year (Fig. 1). Other approaches for assessment of screening utilization were reviewed but not selected in this report (Supplementary Methods).

### Statistical analysis

To test for the effects of CEI, we performed a meta-regression analysis to account for (1) varying population counts behind incidence and mortality empirical estimates (points) and (2) the relationship between these estimates and calendar time due to the evolving population at risk that is partially shared by the points. To perform the meta-regression, we used a linear mixed model with Gaussian random effects adjusted for Race, CEI, SEER Registry, and two- and three-way interactions involving these factors. Unstructured between-point covariance was used while the within-study variances were specified using squared standard errors from the empirical estimates. The model was fit by maximum likelihood^15^.

While any changes in mortality over time in a longitudinal observational study can be explained by an effect of time modeled with sufficient flexibility, such models would be empirical in nature and fail to attribute the effect of time to causal factors. We sought to attribute the dynamics of mortality to a plausible causal factor (CEI or factors independent of CEI) and show that the effect of calendar time could be replaced by the causal factor, provided a good enough explanation of the data was retained. A multivariable model was constructed including CEI per above, as well as switching regression in year 2000 to model the potential effect of any factor or combination of factors that might influence mortality independent of CEI over time starting in 2000 vs prior to 2000. It was surmised that a potential CEI-independent effect of improvement in treatment might be indirectly assessed in this fashion given that SEER data do not include information on treatment that might facilitate a direct assessment of treatment dynamics and mortality improvement in models that also include measures of screening utilization. A sensitivity analysis varying the cutpoint year was conducted to assess the importance of cutpoint selection. Comprehensive data on treatment are not included in SEER and therefore are not available for inclusion in a direct model of treatment effect. Given this reality and the fact that treatment is confounded by screening effects on disease presentation at diagnosis, we elected to avoid biases inherent in complex modeling assumptions that are difficult to test using observational data and pursued the indirect approach described above.

The number of lives saved is calculated as a reduction in mortality weighted by the size of the population and taken cumulative over calendar time. Accumulation (an integral over time) is taken over calendar time t from the year of PSA introduction (1988) to the year of the end of study. A reduction in mortality at time t is the difference between mortality rate without screening (an average over years before 1988) and the mortality rate at time t as predicted by the model. When weighted by the size of the population under study in year t, the reduction in mortality rate (times the population) at t represents the predicted change in the numbers of deaths in the screening era vs. no screening in year t.

## Results

### Overview

Data from 163,982,733 male person-years diagnosed with 544,058 prostate cancers from nine US registries (Atlanta, Connecticut, Detroit, Hawaii, Iowa, New Mexico, San Francisco, Seattle, and Utah) were analyzed. In the eight regions with annual prostate cancer mortality > 10/100,000, the reduction in prostate cancer mortality ranged from 46.0% to 63.7% (Table 1). A total of 27,607 deaths were avoided with mass utilization of PSA screening in the areas assessed (Table 1), representing approximately 9% of the total US population. If extrapolated to the entire US population, the total number of deaths avoided would be 317,356 during the period of PSA screening from 1988 to 2016.

**Table 1:** Mortality reduction and deaths avoided during PSA mass utilization.

Registry	Population (mean)	Baseline annual mortality (deaths/100,000)^a^	Mortality change (%)^b^	Deaths avoided (n)
Atlanta	484,695	62	57.9	3200
Connecticut	699,562	47	60.6	3343
Detroit	758,591	63	63.7	5323
Hawaii	82,541	7	−4.5	−5
Iowa	598,203	52	56.1	3154
New Mexico	333,385	49	46.0	1673
San Francisco	658,664	57	61.9	4206
Seattle	770,131	53	50.9	4494
Utah	353,557	55	56.7	2219

^a^Average of 1980 to 1987.

^b^For years 1988 to 2016.

### Racial variation in prostate cancer mortality reduction

Data from registries where black men represented ≥ 5% of the population (Atlanta, Detroit, San Francisco, and Connecticut) were used to assess the effect of race. White Americans were overrepresented in this data, but on a per-population basis, a greater number of deaths were avoided in the black population (Table 2). Annual prostate cancer mortality was 1.3–2.1 times higher in black than white Americans in all four regions before mass utilization of PSA, with at least double the prostate cancer death rate in black versus white Americans in Atlanta, Detroit, and San Francisco. Racial differences in prostate cancer mortality are also seen when looking at the full SEER dataset (Fig. S1). The percent changes in mortality in the black population with mass utilization of PSA screening ranged from 51.8% in Atlanta to 69.9% in Connecticut compared to 58.8% in Connecticut to 70.0% in Atlanta for white men. Even after mass utilization of PSA, more black men died of prostate cancer in all four registries on a per-population basis.

**Table 2:** Mortality reduction and deaths avoided during PSA mass utilization by race in registries where population was at least 5% black.

Race	Population (mean)	Baseline annual mortality (deaths/100,000)^a^	Mortality change (%)^b^	Deaths avoided (n)	Deaths avoided (per 100,000)
Atlanta					
Black	154,487	111	51.8	1744	1129
White	330,209	54	70.0	2313	700
Connecticut					
Black	49,830	63	69.9	85	170
White	646,434	48	58.8	3069	475
Detroit					
Black	158,062	108	57.4	1717	1087
White	600,529	53	66.4	3712	618
San Francisco					
Black	74,648	103	69.9	925	1239
White	583,879	52	59.3	3266	559

^a^Average of 1980 to 1987.

^b^For years 1988 to 2016 for all groups save for the black population in Connecticut where data are from 1990 to 2016.

### Relationship between CEI and prostate cancer mortality

Having noted substantial declines in prostate cancer mortality, we sought to assess the relationship between these mortality declines and the utilization of PSA screening. After considering multiple approaches for assessment of screening utilization, cumulative excess incidence (CEI) was selected (Supplementary Methods, Fig. 1). This measure serves as a surrogate for screening utilization, as diagnosis requires PSA screening followed by biopsy when appropriate.

**Figure 2:**
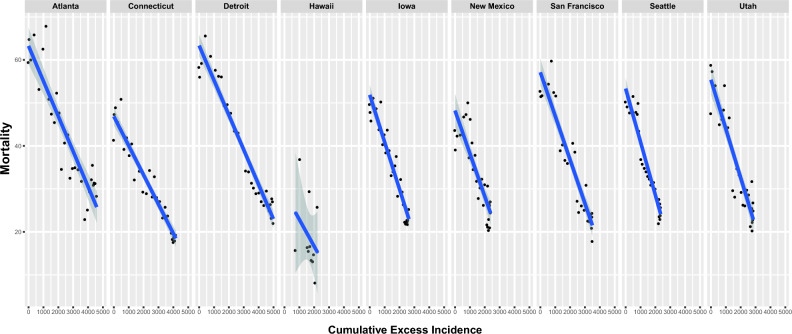
Prostate cancer mortality and CEI by registry. United States state and city population prostate cancer mortality is plotted as a function of prostate cancer CEI from 1988 to 2016.

**Figure 3:**
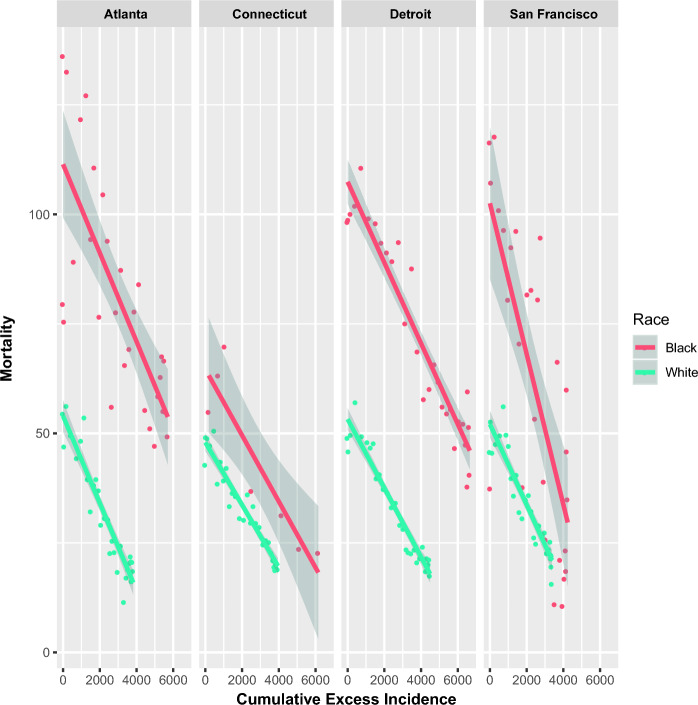
Prostate cancer mortality and CEI stratified by race. Prostate cancer mortality is plotted as a function of prostate cancer CEI stratified by race in more diverse US populations (> 5% black).

**Figure 4:**
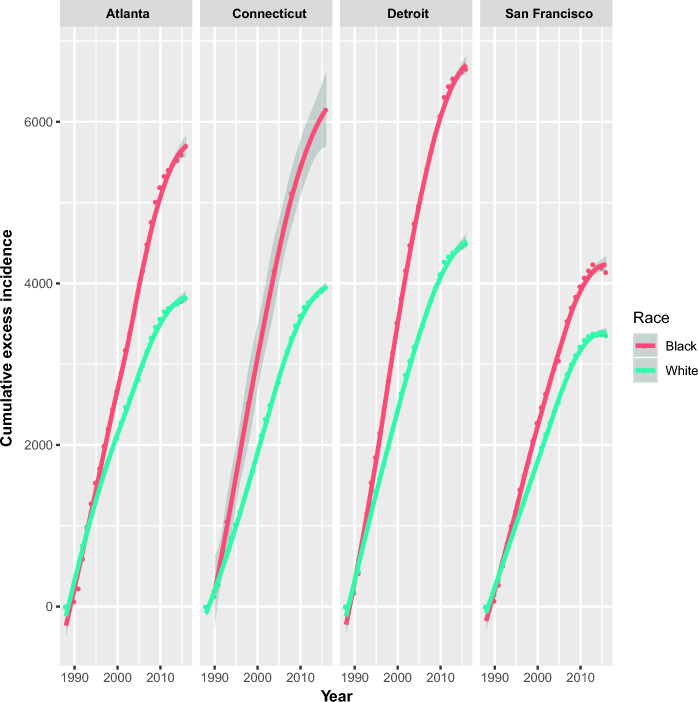
CEI over time stratified by race. Prostate cancer CEI is plotted over time in more diverse US populations (> 5% black).

After the introduction of PSA screening in the US, increased prostate cancer incidence was noted. Registry data demonstrated a decline in prostate cancer mortality with increasing CEI (Fig. 2). A highly significant association between CEI and mortality was noted (p < 0.0001), with mortality reduction of 8.1 (CI: 6.6–9.6) per 1000 units CEI. In both white and black populations, large declines in prostate cancer mortality were noted with increasing CEI (Fig. 3). Mortality tracked with CEI in a linear fashion (Fig. 3). As CEI increased, mortality decreased (Fig. 3). As CEI plateaued, mortality reduction plateaued, maintaining the linear relationship between CEI and mortality reduction (Fig. 3) even as both CEI and mortality changed in a non-linear fashion with time (Fig. 4 and Figure S1, respectively). Additionally, black Americans had much larger CEI than white Americans (by 46.5 (CI:41.7–51.3)). A highly significant effect of the SEER registry persisted throughout, both in the slopes of mortality reduction with CEI as well as the intercepts by registry (p < 0.0001; Fig. 2).

### Relative contribution of factor(s) independent of CEI

Improved cancer treatment has been proposed to explain prostate cancer mortality reduction seen during PSA mass utilization. In order to provide a quantitative assessment of the data to identify a potential effect of treatment improvements or other factors independent of screening, we introduced a possibility of additional improvement due to new treatments starting in year 2000 in the form of an interaction effect between CEI and calendar time (year) > 2000. The additional effect of time (year) in the meta-regression adjusted for CEI did not reach significance. We found that the effect was −0.0011 mortality reduction per year per unit CEI (p-value 0.3357, CI: [−0.0033, 0.0011]). With the highly significant main effect of CEI in this model of −0.0064 (p-value < 0.0001, CI: [−0.0088, −0.0040]), we estimate the relative contribution of factors independent of screening utilization to be 14.6%, not reaching significance. This indicates that factors independent of CEI likely had a small impact on mortality reduction. A sensitivity analysis failed to reach significance for cutpoint years 2000–2010 (p-values > 0.14).

## Discussion

These data demonstrate a strong relationship between CEI (a surrogate for utilization of PSA screening with follow up biopsy) and reduced prostate cancer mortality. Additionally, factors independent of CEI seem to play a small role in the observed mortality reductions. The decline in prostate cancer mortality in areas with baseline mortality > 10/100,000 ranged from 46.0% to 63.7%, seen in both black and white Americans, and 317,356 deaths were prevented. More deaths were avoided among black than white Americans on a per-population basis. These data are cause for hope regarding prostate cancer outcomes and equality in prostate cancer care.

Randomized controlled trials and large retrospective studies examining PSA screening have documented prostate cancer mortality reduction ranging from 0–64% (Table S1). The full ERSPC study demonstrated a 31% mortality reduction with PSA screening after correcting for contamination and compliance^16,17^. In the ERSPC Goteborg subset, biennial screening for 20 years led to a 56% prostate cancer mortality reduction^13^, while in the Rotterdam subset, PSA screening resulted in a 51% reduction with no difference in treatment arms^14^. Similarly, the Kaiser Permanente screening study of over 400,000 men noted 64% reduction in prostate cancer deaths for men age 55–74^18^. Contrary to these results, the PLCO trial initially reported no benefit of organized PSA screening^4,19^. As previously noted, studies to correct for compliance and contamination in this trial have shown a mortality benefit^10,11,20^. Therefore, the mortality reduction seen in this SEER-based population study that is strongly associated with CEI is similar to that seen in multiple randomized and retrospective studies of PSA screening.

An alternative to PSA screening as the explanation for prostate cancer mortality reduction is improved treatment^8^. The relative contributions of screening and treatment to mortality reduction have only been assessed in one prior report to our knowledge^21^. Disentangling relative contributions of screening and improved treatment to mortality reduction over time is challenging. Improvements in prostate cancer treatment occurred simultaneously with increased utilization of screening. Screening is not curative without effective treatment, and treatment options vary with cancer stage at diagnosis. In the absence of screening, more clinically advanced cancers may defeat even improved treatments. Additionally, the progress in prostate cancer therapeutics over time and across the spectrum of early detection is difficult to model because of confounding by indication, insufficient data on specific treatments, and the inability to observe the same patient in both scenarios (with and without early detection).

Despite these complexities, we have attempted to make a quantitative argument as to the effect of treatment or other factors independent of CEI versus the effect of CEI due to the lack of complete collinearity between the utilization of screening and the application of new treatments. In our analysis, we find that the bulk of the mortality reduction seen in the context of PSA mass utilization can be attributed to the effect of screening as assessed by CEI, and the contribution of other factors independent of CEI (including treatment improvement) is only 14.6%, similar to the estimate of treatment effect from Rotterdam of 6%^21^. We do not interpret this analysis to suggest that there is no effect of treatment improvements on mortality reduction. However, in the general population setting, next to the interaction effect of early detection with baseline treatments, treatment improvements represent a relatively small contribution to mortality reduction that fails to reach significance.

Several studies support the position that screening detects cancers at an earlier, more treatable, stage. Prostate cancer mortality reduction with mass utilization of PSA screening was accompanied by a stage shift due to earlier diagnosis^22^. Screening prompted a staging amendment to include non-palpable disease (T1c); this stage, not seen in the pre-PSA era, became dominant^23^. An archived serum study noted that PSA screening could have detected aggressive prostate cancers an average of 5.5 years before clinical diagnosis^24^. At this early stage, prognosis of aggressive cancers is better with treatment^25^. Similarly, a surgical series noted prostate cancer cure rates of approximately 90% in absence of palpable disease and 20% with a palpable nodule^26^. Thus, there are many sources of data that do not support the argument that mortality reduction in the context of PSA mass utilization can be completely explained by improved treatment.

Overdiagnosis and overtreatment refer to the diagnosis and/or treatment of individuals not likely to benefit. When it was thought that PSA screening was unrelated to decreased prostate cancer mortality, recommendations to stop screening^27^ or change screening thresholds^8^ were formulated. Fortunately, approaches are available that permit organized PSA screening while minimizing overdiagnosis and overtreatment^18^. Consideration of age and life expectancy when screening will reduce overdiagnosis^28^. Assessment of patient and disease features when choosing watchful waiting or active surveillance instead of treatment reduces the concern of overtreatment, thereby serving to mitigate the potential harms of screening^29–31^. Further advances in imaging and tumor genetic profiles may improve tumor stratification and further reduce unnecessary interventions^32^. Others have provided multiple arguments suggesting that the impact of overdiagnosis and overtreatment has been overstated^33^.

This is a population study using national databases and is therefore subject to potential unforeseen biases. Data are cross-sectional, limiting ability to follow individuals over the disease course. We have no information regarding treatments that patients received. Lastly, this study is limited to nine US regions and may not be globally representative. The study included data from millions of men with up to 60% or greater level of screening^2^. The study includes an examination of screening in a previously unscreened population, a rarity among screening studies. Due to mass utilization of PSA screening, analysis of historical data from the US may provide the only way to truly assess the relationship between PSA screening and mortality reduction in this population. The study also included registries with substantial black American representation^4^.

In this report, we note strong relationships between CEI in the PSA screening era and mortality reduction. The relationship between factors independent of CEI such as treatment improvements and mortality reduction is much weaker than the relationship between CEI and mortality. During PSA mass utilization, more black than white lives were saved on a per capita basis. Equity in health care delivery is important given the racial disparities in disease outcomes^34^. This work suggests that identifying and obtaining consensus as to the cause of mortality reductions seen during PSA mass utilization is of the utmost importance, as we strive to improve outcomes overall while reducing historical disparities in prostate cancer outcomes. Based on these and other data, the greater hope would be that optimal screening might make symptomatic/fatal prostate cancer presentation largely a relic of the past.

### Supplementary Information

Supplementary Information.

## Data Availability

The datasets analyzed during the current study are available in the Surveillance, Epidemiology, and End Results Program (SEER) repository, https://seer.cancer.gov/data/access.html.
