# Supplementary material for: Mortality reduction and cumulative excess incidence (CEI) in the prostate-specific antigen (PSA) screening era
Source: Sci Rep. 2024 Mar 9;14:5810. doi: 10.1038/s41598-024-55859-z (PMC10925039; doi:10.1038/s41598-024-55859-z)
Supplement: Supplementary file 1 — Supplementary Information. [file 41598_2024_55859_MOESM1_ESM.pdf]

Supplementary Materials

Title: Mortality reduction and cumulative excess incidence (CEI) in the prostate-specific antigen (PSA) screening era

Author List: Patrick W. McLaughlin MD[1,2†], Matthew M. Cousins MD, PhD[1,3†*], Alex Tsodikov PhD[4], Payal D. Soni MD[5], and Juanita M. Crook MD[6]
Affiliations:
[1]Department of Radiation Oncology, University of Michigan, Ann Arbor, MI, USA
[2]Department of Radiation Oncology, Assarian Cancer Center, Ascension Providence Hospital, Novi, MI, USA
[3]Department of Radiation Oncology, Self Regional Healthcare, Greenwood, SC, USA
[4]Department of Biostatistics, University of Michigan School of Public Health, Ann Arbor, MI, USA
[5]Department of Radiation Oncology, Dignity Health Cancer Institute, Phoenix, AZ, USA
[6]British Columbia Cancer Agency and University of British Columbia, Kelowna, British Columbia, Canada

Table of Contents

Item *Page*

Supplementary Methods. Selection of methodology to assess screening utilization ------- 2

Figure S1. Prostate cancer mortality by race --- 3

Table S1. Summary of mortality reduction on cited randomized controlled trials and mass utilization studies --- 4

References --- 6

Supplementary Methods. Selection of methodology to assess screening utilization.

Several metrics were considered for characterization of screening utilization. These included 1) proportion of the population screened with PSA testing, 2) change in proportion of patients with metastatic disease at diagnosis, 3) comparison between population assessments from large databases and the 100% screened arm from PLCO, and 4) cumulative excess incidence.

First, the proportion of the population screened with PSA testing was considered as a way to assess screening utilization; however, this approach met with two major limitations. First, most studies employed Medicare claims to determine whether individuals were subject to PSA testing, restricting the survey to men over 65. Second, PSA testing is only part of full screening (screening is incomplete unless threshold PSA is followed by biopsy). Complete data would include a true screening utilization defined as the percentage of patients who received a PSA testing and a biopsy if indicated based on the PSA test result. The best population estimate for PSA testing alone was ~50% [1].

Second, a decrease in metastasis at diagnosis was considered as a surrogate for screening utilization. Within 10 years of the PSA mass utilization, metastases at diagnosis dropped by approximately 50% in the United States population [2]. One would reasonably surmise that this would require a 50% screening utilization to find cancers prior to metastasis to facilitate a reduction in metastasis at diagnosis of 50%.

Third, the 100% screened arm of PLCO was employed as a surrogate for 100% screening [3]. This also had limitations because in PLCO an elevated PSA only triggered a conversation about biopsy. Biopsy was not mandated as it was in other randomized trials. Also, some men on the screening arm were non-compliant and were not screened. In spite of these limitations, mortality on the 100% screened arm was remarkably low, approximately 30% relative to pre-PSA population mortality. Full reconciliation of mortality reduction on randomized controlled trials vs population mortality reduction is not trivial and is beyond the scope of the current study. Nonetheless, screening arms of randomized studies had marked mortality reduction relative to pre-screening population mortality [4].

In considering the above approaches as ways to understand screening utilization in relation to mortality, valuable insight was gained, but deficiencies that would limit accurate quantification of screening utilization made these approaches less useful for the purpose of assessing the relationship between screening utilization and mortality. Therefore, a fourth approach was considered, cumulative excess incidence (Figure 1). Beginning with the mass utilization of PSA screening, the increased incidence after PSA testing was comprehensively captured as the cumulative incidence, with additional incidence due to screening or “excess” incidence determined by subtracting a curve for baseline incidence (average incidence 1980-1987) from a curve reflecting incidence over time since the PSA mass utilization (Figure 1). In order to diagnose a new patient with prostate cancer (identify a new case), both PSA testing and biopsy would have been required. This means that excess incidence of prostate cancer is a summation of screening effect reflecting the cases that were identified after introduction of PSA screening that would not have been identified without screening.

Conclusion: An approach that allows quantification of the effects of PSA screening with appropriate use of biopsy was identified after considering multiple methods to characterize screening utilization. The use of cumulative excess incidence provides an approach to quantify screening utilization to assess relationships between screening utilization and mortality reduction.

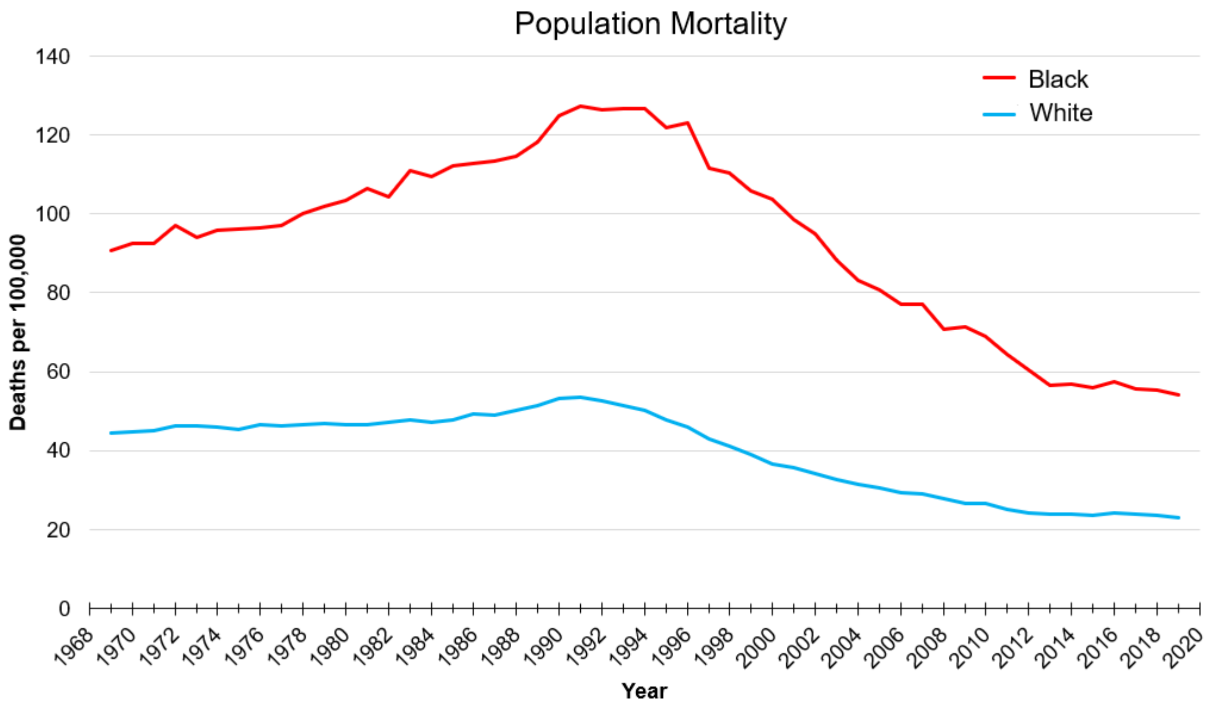

Figure S1. Prostate cancer deaths by race. Annual death rates are shown for black and white American populations as demonstrated by SEER data between 1969 and 2019. Death rates for black Americans are more than 2-fold those of white Americans throughout the entire period of study.

Table S1. Summary of mortality reduction on cited randomized controlled trials and mass utilization studies.

Study Name	Trial Type	Mortality Reduction	Citation[a]
CAP[b]	RCT	0%	[5]
ERSPC[c]	RCT	31%	[6,7]
ERSPC Goteborg[d]	RCT	56%	[8]
ERSPC Rotterdam[e]	RCT	51%	[9]
PLCO[f]	RCT	0%	[3,10]
Kaiser Permanente[g]	Population	64%	[11]
Tyrol, Austria[h]	Population	54%	[12]
Present analysis	Population	46% – 64%	

Abbreviations: CAP – Cluster Randomized Trial of PSA Testing for Prostate Cancer; ERSPC – European Randomized Study of Screening for Prostate Cancer; PLCO – Prostate, Lung, Colorectal and Ovarian Screening Trial; RCT – randomized controlled trial; Population – population study

[a]Reporting entity: Report written by the study team.

[b]CAP: The CAP included more than 415,000 men from the United Kingdom randomized to a single PSA screening event vs no screening and found no difference in prostate cancer specific mortality at 10 years. Only 36% of men in the intervention group had PSA test collected (85% of those meeting biopsy threshold underwent biopsy). Contamination in the control group was estimated at 10-15%.

[c]ERSPC: The ERSPC included more than 160,000 individuals in 8 European countries. Different screening programs were used across participating countries, and a reduction in mortality was observed when data were analyzed in aggregate.

[d]ERSPC Goteborg: ERSPC Goteborg included 20,000 individuals selected from the national registry for Goteborg, Sweden and randomized to screening (10 rounds of biennial PSA screening) or population standard (no formal screening) and demonstrated a mortality reduction of 56% [8]. The approach to recruitment in this trial led to low contamination (3% screened in the control arm) and high compliance (93% compliance with biopsy when indicated) [8]. This trial also noted that the greatest mortality reduction through PSA screening was achieved in those aged 55-59 years [13], direct evidence that age optimization is critical to full mortality reduction. Interestingly, this study also found that opportunistic PSA screening resulted in worse overdiagnosis than organized screening [14].

[e]ERSPC Rotterdam: ERSPC Rotterdam included 40,902 individuals from the Dutch city of Rotterdam randomized to screening (5 rounds of PSA screening conducted every 4 years) versus no formal screening. This study had more contamination (19% screened in the control arm) and non-compliance (5%) than ERSPC Goteborg, but it was possible to correct for these deficits, yielding a 51% mortality reduction [9]. The study also sought to clarify the mechanism of mortality reduction. Men diagnosed later in the disease course on the unscreened arm received superior treatment, including androgen depravation therapy, but superior treatment could not compensate for later diagnosis [15]. The bulk of the mortality benefit (94%) was explained by screening, suggesting that early diagnosis through screening was key to mortality reduction [16].

[f]PLCO: The PLCO included 76,683 individuals from the United States randomized to screening (6 rounds of annual PSA screening) versus opportunistic screening. This trial did not demonstrate a mortality benefit from organized screening [3,10]. However, this trial occurred in the context of the mass utilization of PSA screening in the United States (enrolled 1993-2001), so a large number of individuals in the control arm were screened with PSA (44%) or digital rectal exam (55%) in the three years before the study [3]. By the time longer term follow up data had been collected, 86% of those in the control arm had been screened [17]. Therefore, it is important to note that this trial compared organized and opportunistic screening over a relatively short intervention period. Analyses that have sought to correct for substantial contamination in PLCO have revealed a mortality benefit from PSA screening in this study [18]. Lastly, 15% of the deaths in the screening arm were in men who had never been screened [19]. This suggests that lack of compliance might explain at least a portion of the mortality in the organized screening arm of the trial.

[g]Kaiser Permanente: A retrospective study of 400,887 patients with care managed by Kaiser Permanente who underwent screening with PSA in Northern California during a period of 5 years found substantial reductions in prostate cancer death (64%) and all-cause mortality (24%) in men 55-75 years of age through PSA screening [11]. This study suggested the use of active surveillance to reduce overtreatment and concluded that a screening interval of 1 year was preferred.

[h]Tyrol, Austria: PSA testing was introduced in Tyrol, Austria in 1988 and resulted in death rates that were 54% lower than expected when compared with the rest of Austria [12]. This study noted a greater proportion of disease found at lower stage with PSA screening. In effect, there was localized mass utilization of PSA screening within Tyrol, and this resulted in similar mortality reductions to those seen in the mass utilization of PSA screening in the United States.

References for supplementary methods and Table S1

1 Legler, J. M., Feuer, E. J., Potosky, A. L., Merrill, R. M. & Kramer, B. S. The role of prostate-specific antigen (PSA) testing patterns in the recent prostate cancer incidence decline in the United States. *Cancer Causes and Control* **9**, 519-527 (1998).

2 Chu, K. C., Tarone, R. E. & Freeman, H. P. Trends in prostate cancer mortality among black men and white men in the United States. *Cancer* **97**, 1507-1516 (2003). https://doi.org:10.1002/cncr.11212

3 Andriole, G. L. *et al.* Mortality results from a randomized prostate-cancer screening trial. *N Engl J Med* **360**, 1310-1319 (2009). https://doi.org:10.1056/NEJMoa0810696

4 Shoag, J. E., Nyame, Y. A., Gulati, R., Etzioni, R. & Hu, J. C. Reconsidering the Trade-offs of Prostate Cancer Screening. *N Engl J Med* **382**, 2465-2468 (2020). https://doi.org:10.1056/NEJMsb2000250

5 Martin, R. M. *et al.* Effect of a Low-Intensity PSA-Based Screening Intervention on Prostate Cancer Mortality: The CAP Randomized Clinical Trial. *Jama* **319**, 883-895 (2018). https://doi.org:10.1001/jama.2018.0154

6 Schroder, F. H. *et al.* Screening and prostate cancer mortality: results of the European Randomised Study of Screening for Prostate Cancer (ERSPC) at 13 years of follow-up. *Lancet* **384**, 2027-2035 (2014). https://doi.org:10.1016/S0140-6736(14)60525-0

7 Roobol, M. J. *et al.* Prostate cancer mortality reduction by prostate-specific antigen-based screening adjusted for nonattendance and contamination in the European Randomised Study of Screening for Prostate Cancer (ERSPC). *European urology* **56**, 584-591 (2009). https://doi.org:10.1016/j.eururo.2009.07.018

8 Hugosson, J. *et al.* Mortality results from the Göteborg randomised population-based prostate-cancer screening trial. *The Lancet Oncology* **11**, 725-732 (2010). https://doi.org:10.1016/s1470-2045(10)70146-7

9 Bokhorst, L. P. *et al.* Prostate-specific antigen-based prostate cancer screening: reduction of prostate cancer mortality after correction for nonattendance and contamination in the Rotterdam section of the European Randomized Study of Screening for Prostate Cancer. *European urology* **65**, 329-336 (2014). https://doi.org:10.1016/j.eururo.2013.08.005

10 Andriole, G. L. *et al.* Prostate cancer screening in the randomized Prostate, Lung, Colorectal, and Ovarian Cancer Screening Trial: mortality results after 13 years of follow-up. *Journal of the National Cancer Institute* **104**, 125-132 (2012). https://doi.org:10.1093/jnci/djr500

11 Alpert, P. F. New Evidence for the Benefit of Prostate-specific Antigen Screening: Data From 400,887 Kaiser Permanente Patients. *Urology* **118**, 119-126 (2018). https://doi.org:10.1016/j.urology.2018.02.049

12 Bartsch, G. *et al.* Tyrol Prostate Cancer Demonstration Project: early detection, treatment, outcome, incidence and mortality. *BJU International* **101**, 809-816 (2008). https://doi.org:https://doi.org/10.1111/j.1464-410X.2008.07502.x

13 Hugosson, J. *et al.* Eighteen-year follow-up of the Göteborg Randomized Population-based Prostate Cancer Screening Trial: effect of sociodemographic variables on participation, prostate cancer incidence and mortality. *Scand J Urol* **52**, 27-37 (2018). https://doi.org:10.1080/21681805.2017.1411392

14 Arnsrud Godtman, R., Holmberg, E., Lilja, H., Stranne, J. & Hugosson, J. Opportunistic testing versus organized prostate-specific antigen screening: outcome after 18 years in the Goteborg randomized population-based prostate cancer screening trial. *European urology* **68**, 354-360 (2015). https://doi.org:10.1016/j.eururo.2014.12.006

15 Bokhorst, L. P. *et al.* Differences in Treatment and Outcome After Treatment with Curative Intent in the Screening and Control Arms of the ERSPC Rotterdam. *European urology* **68**, 179-182 (2015). https://doi.org:10.1016/j.eururo.2014.10.008
16 Bokhorst, L. P. *et al.* Do Treatment Differences between Arms Affect the Main Outcome of ERSPC Rotterdam? *J Urol* **194**, 336-342 (2015). https://doi.org:10.1016/j.juro.2015.02.045
17 Pinsky, P. F. *et al.* Extended mortality results for prostate cancer screening in the PLCO trial with median follow-up of 15 years. *Cancer* **123**, 592-599 (2017). https://doi.org:10.1002/cncr.30474
18 Tsodikov, A. *et al.* Reconciling the Effects of Screening on Prostate Cancer Mortality in the ERSPC and PLCO Trials. *Annals of internal medicine* **167**, 449-455 (2017). https://doi.org:10.7326/M16-2586
19 Shoag, J. *et al.* Lethal Prostate Cancer in the PLCO Cancer Screening Trial. *European urology* **70**, 2-5 (2016). https://doi.org:10.1016/j.eururo.2016.01.009
